# Research on lncRNA related to drought resistance of Shanlan upland rice

**DOI:** 10.1186/s12864-022-08546-0

**Published:** 2022-04-30

**Authors:** Xinsen Yang, Caiyue Liu, Xiaoling Niu, Liu Wang, Laiyi Li, Qianhua Yuan, Xinwu Pei

**Affiliations:** 1 Hainan Key Laboratory for Sustainable Utilization of Tropical Bio-Resources, College of Tropical Crops, Hainan University, Haikou, 570228 China; 2 Biotechnology Research Institute, Chinese Academy of Agricultural Sciences, Beijing, 100081 China

**Keywords:** lncRNA, ceRNA, Drought, Shanlan upland rice

## Abstract

**Background:**

Drought has become the major abiotic stress that causes losses in rice yields and consequently is one of the main environmental factors threatening food security. Long non-coding RNA (lncRNA) is known to play an important role in plant response to drought stress, while the mechanisms of competing endogenous RNA (ceRNA) in drought resistance in upland rice have been rarely reported.

**Results:**

In our study, a total of 191 lncRNAs, 2115 mRNAs and 32 miRNAs (microRNAs) were found by strand-specific sequencing and small RNA sequencing to be differentially expressed in drought-stressed rice. Functional analysis of results indicate that they play important roles in hormone signal transduction, chlorophyll synthesis, protein synthesis and other pathways. Construction of a ceRNA network revealed that MSTRG.28732.3 may interact with miR171 in the chlorophyll biosynthesis pathway and affect the ability of plants to withstand drought stress by regulating Os02g0662700, Os02g0663100 and Os06g0105350. The accuracy of the regulatory network was verified by qRT-PCR.

**Conclusion:**

Our results provide a theoretical basis for future studies on the potential function of lncRNA in plant drought resistance, and they provide new genetic resources for drought-resistant rice breeding.

**Supplementary Information:**

The online version contains supplementary material available at 10.1186/s12864-022-08546-0.

## Background

With the influence of global warming, frequent occurrences of extreme climate and water resource shortages, drought has become the strongest abiotic stress causing the greatest losses in rice yield. Drought affects plant growth and development, physiological metabolism, geographical distribution, and quality and yield [1]. Thus, it is one of the main environmental factors threatening food security. When plants suffer from drought, a series of problems, such as detrimental leaf and root morphological changes, material absorption imbalance and reduced photosynthetic capacity, will occur [2–4]. Shanlan upland rice is a unique, local upland rice grown in Hainan Province, China. This rice is characterized by heat and drought resistances [5]. Comparison of phenotypic data and physiological indicators at the seedling stage between Shanlan upland rice and common cultivated rice under drought stress showed that Shanlan upland rice had stronger drought resistance [6]. The drought resistance mechanism of plants is very complicated. Plants with high water retention rates *in vitro* and strong plasma membrane stability are more resistant to drought. Under dry farming conditions, the leaf water retention rate and the stability of plasma membrane of Shanlan upland rice are higher than those of cultivated rice [7]. Liu et al. [8] used gene chip technology to study the gene expression profile of drought stress in Shanlan upland rice, and they found more than 1700 genes responding to drought stress. Moreover, after screening OsMS17 for overexpression in the cultivar Nipponbare, their results showed that drought resistance of OsMS17 transgenic plants was significantly enhanced at the seedling and maturity stages. Therefore, it is of great significance to study the drought resistance mechanism of Shanlan upland rice for the cultivation of drought-resistant rice and the development of water-saving rice production.

Long non-coding RNA (lncRNA), commonly defined as an RNA transcript that is more than 200 nucleotides in length and has no coding ability [9], can regulate target genes through cis-acting and trans-acting elements [10]. Cis-acting elements function in transcriptional activation or expression regulation of adjacent mRNA with lncRNA. Trans-acting lncRNA can affect genes at many distant sites by binding enhancers or promoters. Early studies questioned the importance of lncRNA for several reasons. Compared with mRNA, lncRNA has been characterized by low expression levels and low conservation of sequence. Additionally, it was considered as a by-product of RNA polymerase II transcription, so its existence was attributed to “transcriptional noise” [11]. With the progress made by non-coding RNA research, the unique biological characteristics and functions of lncRNA have been identified, and a large number of studies have shown that lncRNA is related to drought stress response. Liu et al. [12] treated *Arabidopsis thaliana* with drought stress and abscisic acid (ABA) and found that the expression of At5NC05682 changed significantly after the treatments. Thus, researchers have speculated that lncRNA may play a role in plant resistance to drought stress. Qin et al. [13] discovered a new drought-induced lncRNA (DRIR), which can be activated after drought, salt stress and ABA treatments. Transcriptome analysis of DRIR mutants showed that DRIR can improve the tolerance of plants to drought and salt stresses by regulating ABA signaling, water transport and genes related to stress resistance. Li et al. [14] identified a large number of genes related to drought “memory” in rice by strand-specific sequencing and demonstrated that rice can form drought “memory” in response to repeated drought stress. They found that both lncRNA and plant hormones (especially ABA) are involved in the formation of short-term drought “memory” and activate the expression of “memory” transcripts in metabolic pathways, such as photosynthesis and proline synthesis, thus improving the ability of plants to resist drought stress. Lu et al. [9] used RNA-seq technology in cotton (*Gossypium hirsutum*) to study the role of lncRNA in response to drought stress, and they found that two lncRNAs, XLOC_063105 and XLOC_115463, may be involved in regulating the response pathways of plant hormones to drought stress. Tao et al. [15] found that the long intergenic lncRNA (lincRNA) GhDAN1 in an allotetraploid upland cotton can inhibit the AAAG-containing gene in the auxin response pathway, thereby affecting the drought resistance of the plant. All of these studies suggest that lncRNA plays a role in plant resistance to drought stress.

The concept of ceRNA (competing endogenous RNA), proposed by Salmena et al. [16], was described as miRNA targets (circRNA, lncRNA, mRNA and pseudogenes, etc.) competing with common microRNA response elements (MREs) to induce indirect regulation. The first example in plants was discovered in *A. thaliana*. Researchers found that non-coding RNA IPS1 can affect the expression of PHO2 by binding miR399; they define this mechanism as “target mimicry” [17]. More recent studies have also found that lncRNA can function as ceRNA. By constructing a ceRNA network, Chen et al. [18] found that overexpression of lncRNA TCONS 00,021,861 weakened the inhibitory effect of miR528-3p on YUCCA7 and increased the content of indoleacetic acid (IAA) in rice. Li et al. [19] used RNA-seq and degradation sequencing technology to study the differentially expressed mRNA, miRNA, lncRNA and circular RNA (circRNA) in leaves and roots of sugar beet under salt stress. They revealed that response of the ceRNA regulatory network to salt stress is related to copper redistribution, plasma membrane permeability, glucose and energy metabolisms, NAC transcription factors and the phosphoinositol signaling system. He et al. [20] predicted that lncRNA TCON_00031790, TCON_00014332, TCON_00014717 and TCON_00005674 along with miR9748 play an important role in cucumber response to high temperature stress by regulating plant hormone signaling pathways. Yang et al. [21] found that lncRNA could regulate mRNA expression at the flowering stage by forming ceRNA and then further regulate a variety of biological metabolic pathways of tomato. Thus, research to date suggests that lncRNA not only play an important role in stress resistance but also in plant growth and development as ceRNA.

“Shanchuan 24” is a variety of Shanlan upland rice and has stability in heritability, a developed root system and strong drought resistance. Our team has studied changes of source-sink-translocation in “Shanchuan 24” under different cultivation methods [22] and the effects of different nitrogen application rates on photosynthesis and yield of this variety [23]. In this study, “Shanchuan 24” was used to study the mechanism of drought resistance of Shanlan upland rice at the molecular level. Strand-specific sequencing and small RNA sequencing data obtained from leaves were compared between a drought-stress treatment and a non-stressed control group. lncRNA, miRNA and mRNA related to drought resistance were screened and a ceRNA network was constructed. The potential regulatory effects of theses RNAs were analyzed by Gene Ontology (GO) enrichment and Kyoto Encyclopedia of Genes and Genomes (KEGG) analyses. A ceRNA network was used to elucidate the potential functions and analyze the potential regulatory mechanism of lncRNA. Our results provide a theoretical basis for future studies on the function of lncRNA in plant drought resistance.

## Results

### Identification of lncRNA, miRNA and mRNA

**Fig. 1:**
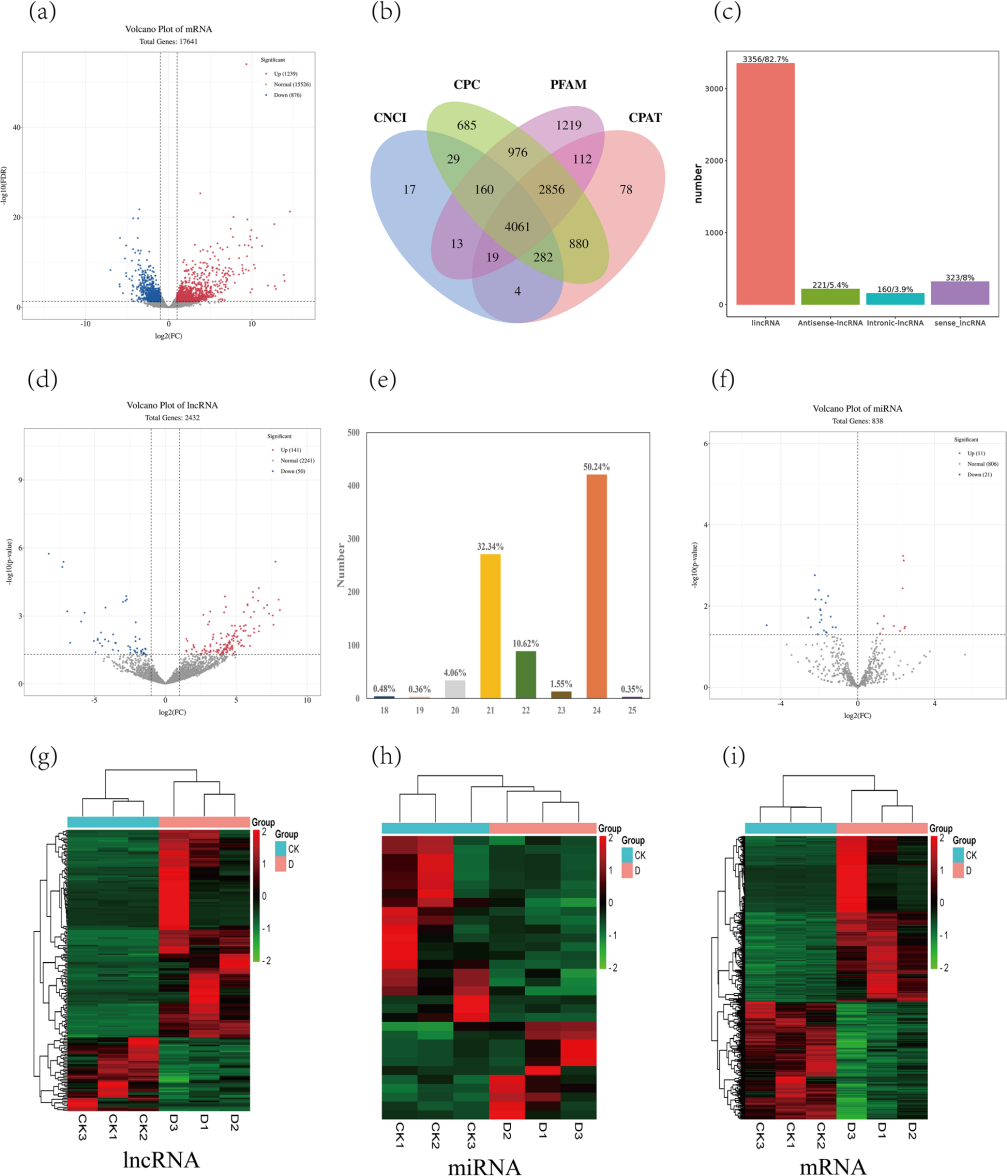
Identification of lncRNA, miRNA and mRNA. **a**: Volcanic diagram of mRNA. **b**: The Venn diagram drawn by the results of CPC, CNCI, CPAT and PFAM. **c**: lncRNA category diagram. **d**: volcanic diagram of lncRNA. **e**: Length histogram of all the miRNAs identified in the samples. **f**: Volcanic diagram of miRNA. **g**, **h**, **I**: Heat maps of differentially expressed lncRNA, miRNA and mRNA under drought stress

We constructed six cDNA libraries (CK1, CK2, CK3 and D1, D2, D3). A total of 333,005,017 raw reads were obtained by strand-specific transcriptome sequencing, and each sample had more than 50,000,000 raw reads. After filtering raw reads, the numbers of clean reads obtained from CK1, CK2, CK3, D1, D2 and D3 samples were 53,082,927, 49,789,943, 51,376,996, 57,970,518, 51,301,419 and 64,833,793, respectively. The Q20 values of clean reads were greater than 97%, the Q30 values of all samples were greater than 91%, and the GC contents were greater than 46%. These values indicate the high quality of our clean reads (Table 1). We aligned the clean reads of each sample with the reference genome and obtained 5,131 mRNAs. Gene expression was spatio-temporal specific, and external stimuli and the internal environment could affect gene expression. In total, we identified 2,115 differentially expressed mRNAs of which 1,239 were up-regulated and 876 were down-regulated (Fig. 1a). In addition, four genes that specifically expressed in plants under drought were screened for, among which Os01g0910800 was the most significantly up-regulated, followed by Os02g0160400, Os05g0586700 and Os07g0188700.

**Table 1:** Data of strand-specific transcriptome sequencing

Sample	Raw Reads	Clean Reads	Q20(%)	Q30(%)	GC Content (%)
CK1	53,758,592	53,082,927	97.8	93.84	47.83
CK2	50,332,480	49,789,943	97	91.85	47.53
CK3	51,882,306	51,376,996	97.56	92.96	46.16
D1	59,023,005	57,970,518	97.75	93.72	47.89
D2	52,043,555	51,301,419	97.45	93.01	47.61
D3	65,965,079	64,833,793	97.56	93.41	49.26

For lncRNA, after basic screening, the transcripts without coding ability were screened using CPC, CNCI, and CPAT and PFAM. We identified a total of 54,880 lncRNAs among the six cDNA libraries where 15,812 were identified by CPC analysis, 15,762 by CNCI analysis, 8,291 by CPAT analysis, and 15,015 by PFAM analysis (Fig. 1b). Each of the four methods identified the same 4,060 lncRNAs and so these were considered as new lncRNAs. Among these new lncRNAs 3,356 (82.7%) were located in intergene regions, so we classified them as lincRNA. These accounted for the largest proportion of new lncRNAs, while 160 intronic-lncRNAs accounted for 3.9% of the new lncRNAs. There were 323 sense-lncRNAs, accounting for 8%, and 221 antisense-lncRNAs, accounting for only 5.4% of the new lncRNAs (Fig. 1c). Of the drought stress treatment, a total of 191 differentially-expressed lncRNAs were obtained, among which 141 were up-regulated and 50 were down-regulated (Fig. 1d, supplemental Table S00^4^). Among the differentially-expressed lncRNAs, 60 lncRNAs were specifically expressed under drought conditions (supplemental Table S00^1^). Among them, the most significant expression levels were observed in MSTRG.5679.8, MSTRG.19712.1 and MSTRG.37152.2. In addition, 10 lncRNAs were specifically expressed in the control group, where the most significantly down-regulated lncRNA was MSTRG.18092.1, followed by MSTRG.40822.2 and MSTRG.27106.6 (supplemental Table S00^2^).

We completed small RNA sequencing of the six samples (CK1, CK2, CK3 and D1, D2, D3) of rice leaves at the three-leaf stage which resulted in a total of 61,665,353 clean reads. Each sample had more than 9,600,000 clean reads (Table 2). After screening, we obtained 838 miRNAs consisting of 430 known miRNAs and 408 newly predicted miRNAs, most of which were 21 nt (32.34%) and 24 nt (50.24%) in length (Fig. 1e). Under drought stress, 32 miRNAs were differentially expressed, among which 11 were up-regulated and 21 were down-regulated (Fig. 1f, supplemental Table S00^5^). Family analysis of these miRNAs identified more of them in the miR171 family than in any other identified family (supplemental Table S00^5^).

**Table 2:** Data of small RNA sequencing

Sample	Raw Reads	Clean Reads	Q20(%)	Q30(%)	GC Content (%)
CK1	11,375,030	10,985,113	99.4	97.89	52.56
CK2	10,776,389	10,097,213	99.16	97.38	52.69
CK3	10,974,474	10,749,889	99.22	97.36	52.85
D1	10,023,828	9,873,147	99.47	98.07	53.37
D2	9,963,724	9,664,232	99.37	97.78	52.56
D3	10,714,065	10,295,759	99.37	97.53	53.19

Hierarchical cluster analysis was performed on the differentially expressed lncRNAs, miRNAs and mRNAs, and the RNAs with the same or similar expression behavior were clustered. The clustering results are shown in Fig. 1g, h, and i.

### Comparative analysis of lncRNA and mRNA

**Fig. 2:**
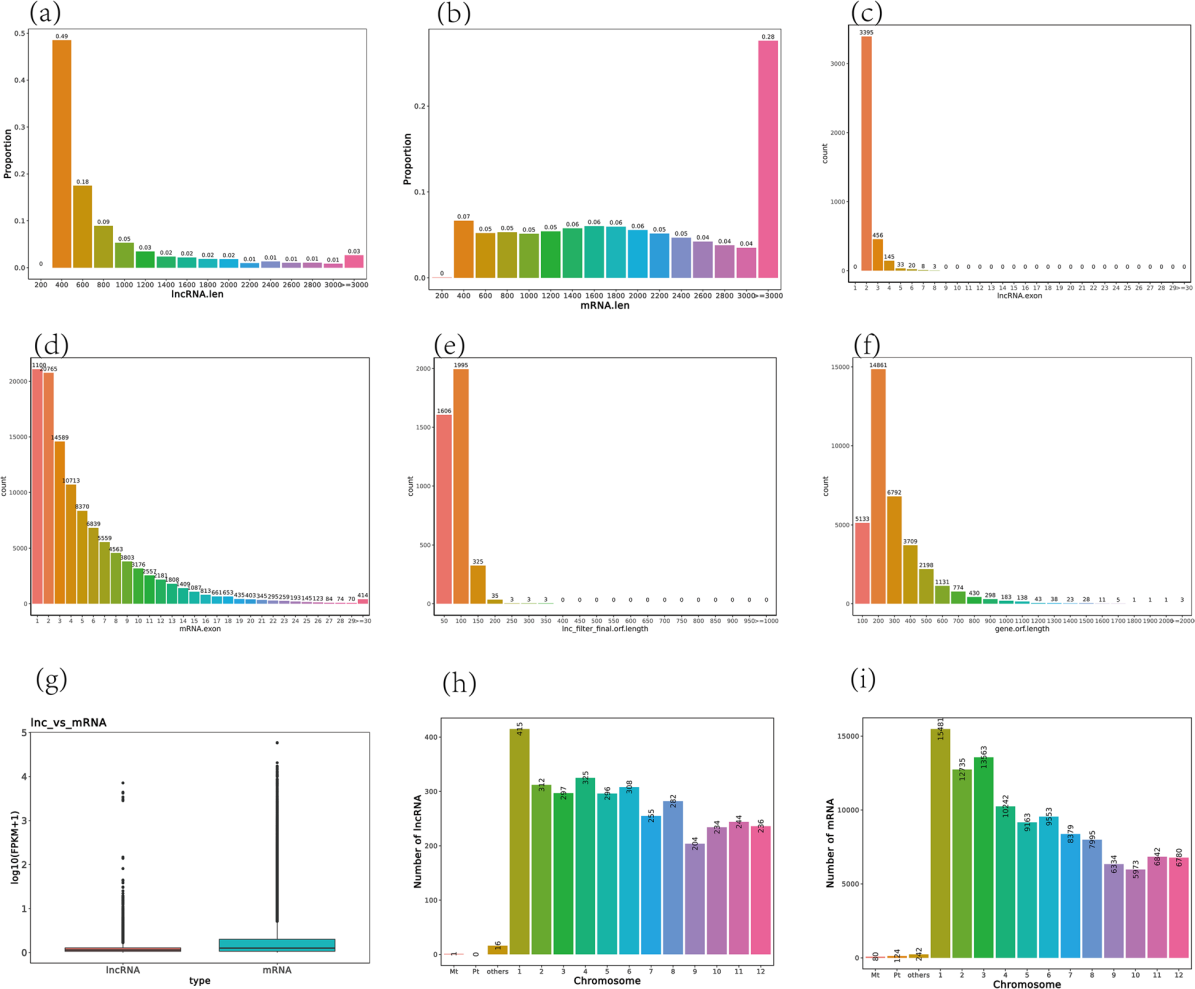
Comparative analysis between lncRNA and mRNA. **a**, **b**: Sequence length diagram of lncRNA and mRNA. **c**, **d**: Exon number of lncRNA and mRNA. **e**, **f**: ORF lengths of lncRNA and mRNA. **g**: comparison of lncRNA and mRNA expression levels. **h**, **i**: Distribution characteristics of lncRNA and mRNA, respectively. 1 ~ 12 correspond to 12 pairs of chromosomes. Mt represents mitochondria and Pt represents chloroplast

We compared the sequence characteristics of the identified lncRNA and mRNA. The lncRNAs that were 300–500 bp in length accounted for 48.55% of the identified lncRNAs, while the mRNAs greater than 1900 bp accounted for 54.50% of the identified mRNAs (Fig. 2a, b). The average mRNA sequence was much longer than the average lncRNA sequence. The number of exons in each lncRNA did not exceed eight among all lncRNAs. Approximately 83.62% of lncRNAs had two exons, while 63.11% of mRNA had more than two exons (Fig. 2c, d). The largest number of lncRNAs had an ORF length of roughly 100 bp, while the largest number of mRNAs had an ORF length of about 200 bp (Fig. 2e, f). In addition, we analyzed the expression levels of lncRNA and mRNA, and found that the FPKM of lncRNA was lower than that of mRNA (Fig. 2g). Moreover, the numbers of lncRNA and mRNA present in chromosomes were distributed differently among the different chromosomes. However, their trends in distribution were similar by chromosome, for example, the highest numbers of lncRNA and mRNA were observed in the first chromosome (Fig. 2h, i).

### Target gene prediction and functional annotation

lncRNA can regulate the expression of its adjacent genes and also act on further genes through base complementary pairing. We predicted 3,284 lncRNA cis-target genes within 100 kb upstream and downstream of 192 differentially-expressed lncRNAs. According to the principle of base complementary pairing, we predicted 574,491 lncRNA trans-target genes (supplemental Table S00^4^). In plants, miRNA and target genes complement each other almost perfectly, and miRNA regulate the expression of target genes by inhibiting translation. Because of the almost-perfect complementarity, we were able to predict 793 target genes with differential expression of miRNA (supplemental Table S00^5^).

**Fig. 3:**
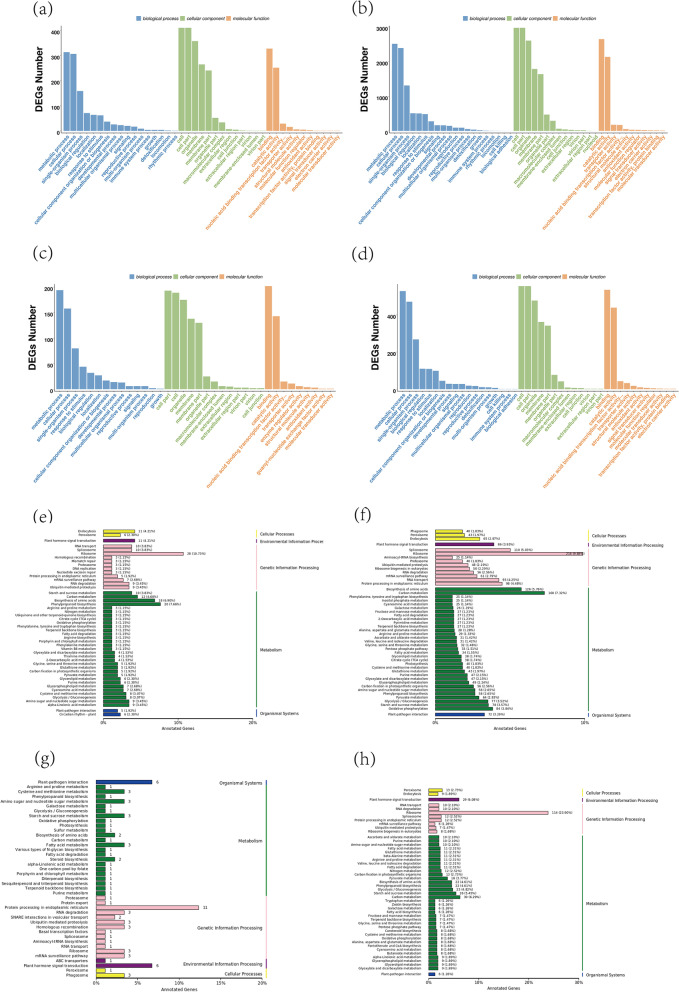
Functional analysis of differentially expressed lncRNA, miRNA and mRNA under drought stress. **a**, **b**, **c**, **d**: GO enrichment analysis of lncRNA cis-target genes, lncRNA trans-target genes, miRNA target genes and mRNA, respectively. **e**, **f**, **g**, **h**: KEGG pathway analysis of lncRNA cis-target genes, lncRNA trans-target genes, miRNA target genes and mRNA, respectively

In order to explore the functions of lncRNA, miRNA and mRNA in response to drought stress, we conducted GO functional enrichment analysis and KEGG pathway analysis of differential genes under drought stress (Supplemental tablesS00^6^, S00^7^, S00^8^, and S00^9^). According to GO functional enrichment, most differentially expressed lncRNAs, miRNAs and mRNAs annotated to the same function. Among biological processes, the largest number of annotated genes were metabolic processes (GO:0,008,152). Moreover, cellular process (GO:0,009,987), single-organism process (GO:0,044,699), response to stimulus (GO:0,050,896) and biological regulation (GO:0,065,007) were also noted. Most of the differentially-expressed genes in cell components were annotated in terms related to membrane. Among terms in the category of molecular functions, binding (GO:0,005,488), catalytic activity (GO:0,003,824), nucleic acid binding transcription factor activity (GO:0,001,071), and transport activity (GO:0,005,215) had the largest numbers of differential genes. KEGG pathway analysis showed that the pathways annotated by the three types of differentially-expressed RNAs were assigned to five main categories, metabolism, genetic information process, environment information process, cellular process and organismal systems. We found that a large number of lncRNA cis-target genes and trans-target genes annotated to ribosomal-related pathways. In addition, the pathways annotated of three RNA types were roughly the same, with a large number of differentially-expressed genes involved in plant metabolic activities. Pathways such as porphyrin and chlorophyll metabolism, carbon metabolism, cysteine and methionine metabolism, fatty acid degradation, photosynthesis, arginine and proline metabolism related to plant drought resistance were all annotated (Fig. 3).

### Construction of ceRNA network related to drought resistance

**Fig. 4:**
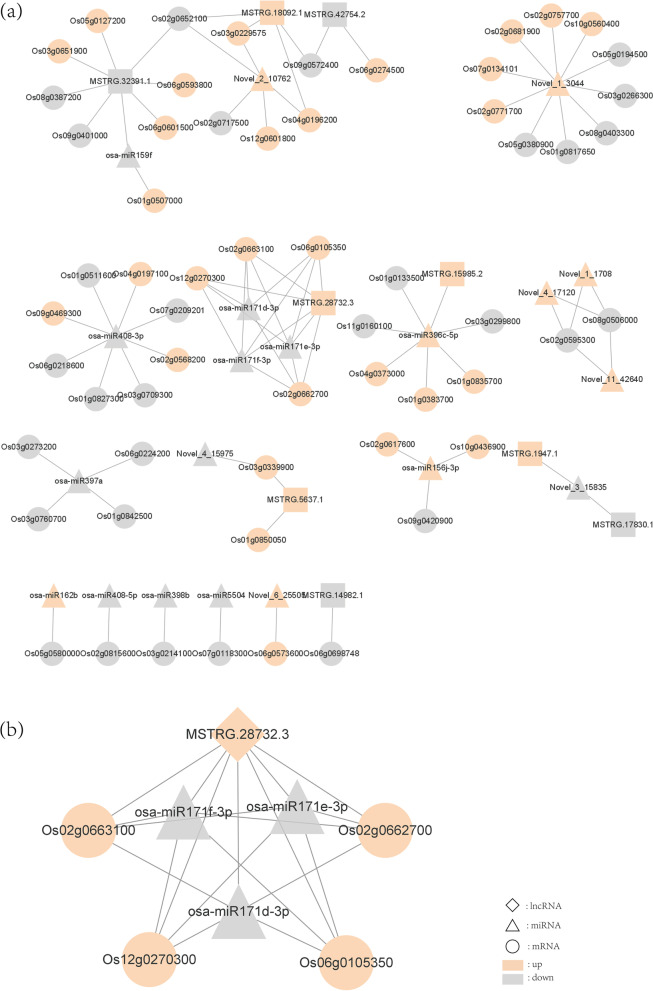
ceRNA network in Shanlan upland rice under drought stress. **a**: A panoramic network composed of 9 lncRNAs, 20 miRNAs and 59 mRNAs. **b** One lncRNA, three miRNAs and four mRNAs constituted the subnetwork

Using TargetFinder to perform sequence comparisons of all types of RNA obtained by sequencing, we found relationships of 742 lncRNAs and 12,164 mRNAs with 777 miRNAs (supplemental Table S00^10^). According to ceRNA-score principle, 675 lncRNA-mRNA pairs were screened (supplemental Table S00^11^). Then, differentially expressed RNAs identified in rice under drought were screened, and we obtained 61 miRNA-mRNA, 7 lncRNA-miRNA and 20 lncRNA-mRNA pairs, all of which were composed of 9 lncRNAs, 20 miRNAs and 59 mRNAs. Cytoscape3.8.2 software was used to construct a regulatory network diagram (Fig. 4a). After screening for relationships of lncRNAs, miRNAs and mRNAs, 16 networks composed of one lncRNA (MSTRG. 28,732.3), three miRNAs (osa-miR171d-3p, osa-miR171e-3p, osa-miR171f-3p) and four mRNAs (Os02g0662700, Os02g0663100, Os06g0105350, os12g0270300) were obtained (Fig. 4b). The three miRNAs in the 16 networks belong to the miR171 family and they all fall into one subcategory [24]. Target genes Os02g0662700, Os02g0663100, and Os06g0105350 of the four mRNAs annotated as straw-like protein 6 (SCL 6) based on NCBI non-redundant protein sequence (NR) annotations. Os12g0270300 annotated as disease resistance protein RGA5-like (supplemental Table S00^9^).

### ceRNA network was verified by qRT-PCR

**Fig. 5:**
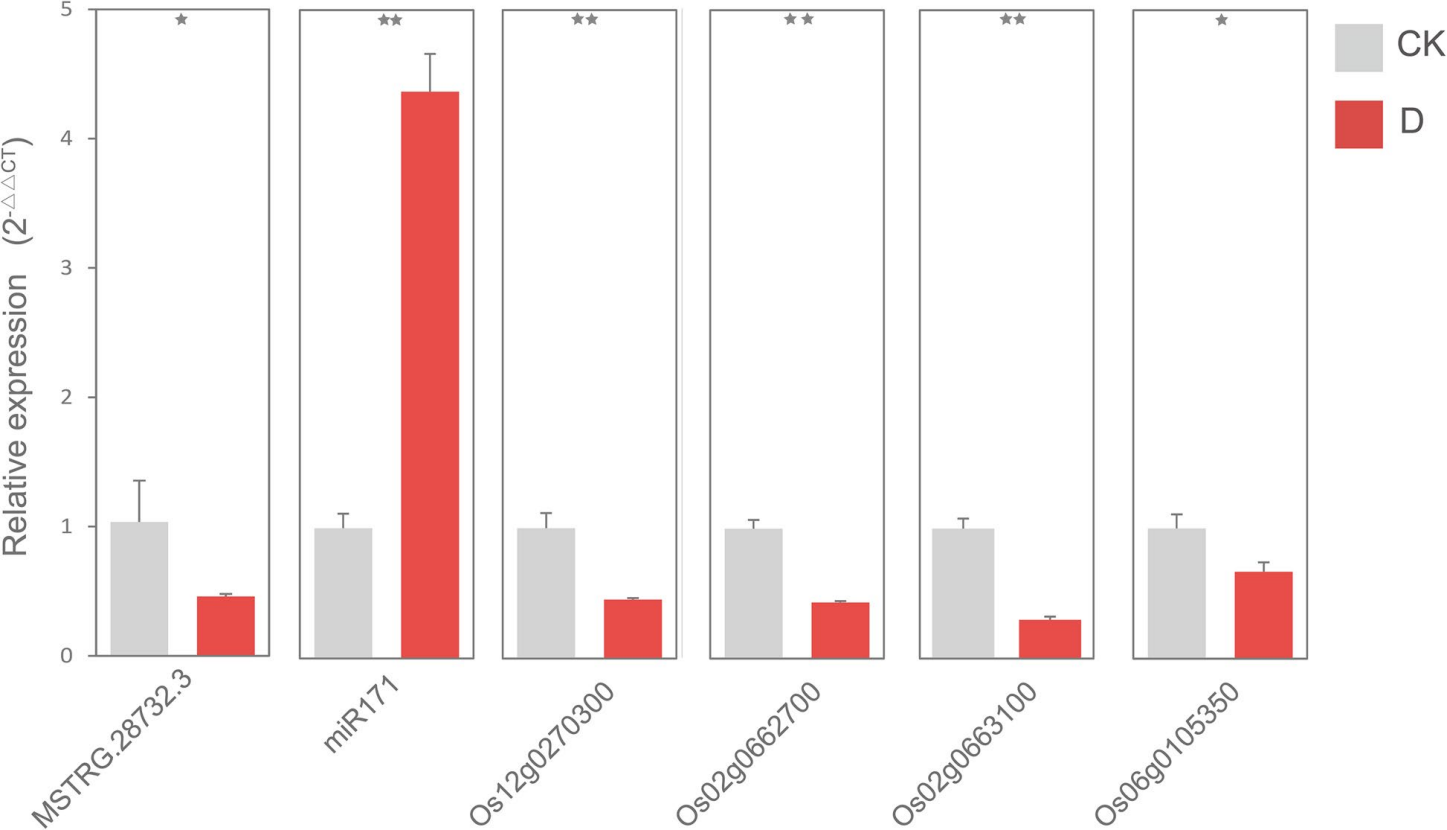
qRT-PCR results of RNA in ceRNA network. Red represents the drought treatment group and gray represents the control group. On the X-axis are the different RNAs, and on the Y-axis are the relative expression levels calculated using the 2^−△△CT^ method. A star on top of each bar indicates statistically significant difference (*p* < 0.05). Two stars indicate extremely significant difference (*p* < 0.01)

To confirm the quality of lncRNA, miRNA and mRNA sequencing and expression patterns in the control and drought groups, we used qRT-PCR to analyze the relative expression levels of MSTRG.28732.3, miR171, Os02g0662700, Os02g0663100, Os06g0105350 and Os12g0270300 in the ceRNA network. Shown in Fig. 5, drought stress up-regulated miR171 expression by 4.3 times and down-regulated lncRNA (MSTRG.28732.3) expression by 2.3 times that of their respective controls. Four genes (Os02g0662700, Os02g0663100, Os06g0105350, and Os12g0270300) were down-regulated by 2.2, 2.3, 3.3 and 1.4 times, respectively. Among them, result of Os12g0270300 was significantly different from the 6.2 times of the sequencing result. We speculate that the difference may be caused by differences between samples. On the whole, trends observed in these results were similar to trends in the transcriptome data, indicating that our sequencing results are reliable and bioinformatics data analysis could be continued.

## Discussion

A large number of studies have shown that lncRNA, as ceRNA, can play an important role in plant growth and development and stress resistance by competing with miRNA targets and binding MREs [18, 19, 21]. Through the ceRNA network formed using the lncRNA, miRNA and mRNA data, we have a clearer understanding of regulatory mechanisms of expression among genes at the transcriptional level. Although a large number of lncRNAs have been identified in plants, the functions of most lncRNAs have not been fully understood. In order to explore the function of lncRNA in drought stress, we used high-throughput sequencing technology to analyze the transcriptome of lncRNA, miRNA, and mRNA in Shanlan upland rice, a characteristic Hainan upland rice.

A total of 4,060 lncRNAs were identified in the leaves of Shanlan upland rice. Similar to previous studies, most lncRNAs were classified as lincRNA [25]. We compared the basic characteristics of lncRNA and mRNA and found that the lengths of lncRNA and ORF sequences were shorter than lengths of mRNA sequences. In addition, lncRNA had fewer exons and lower expression levels, which is in line with previous research results [18, 26].

Compared with the control group, 191 differentially-expressed lncRNAs were found in rice under drought stress, of which 141 were up-regulated and 50 were down-regulated. In order to understand the function of lncRNAs, we predicted the target genes of the 191 differentially-expressed lncRNAs. The function analysis revealed that the target gene were enriched in pathways associated with photosynthesis and hormone metabolism. This analysis indicates that drought stress had a great impact on plant hormone metabolism and photosynthesis, which is consistent with analyses of previous studies [27]. Generally, lncRNAs lack the ability to encode proteins. However, we found that a large number of lncRNAs annotated to the pathway related to ribosomes. Plants subjected to drought stress will respond with changes in expression of a large number of genes, and protein synthesis occurs in the ribosome. Thus, we speculate that although lncRNAs do not encode proteins, they regulate gene expression at the transcriptional level in ways unknown.

Membranes play an important role in material transport and osmotic regulation in plant resistance to drought stress [28, 29]. When plants are subjected to drought stress, the amount of reactive oxygen species (ROS) increases significantly in plants [30], and high concentration of ROS can lead to lipid oxidative stress [31] that will damage plant membranes. In our study, we found 60 out of 191 differentially-expressed lncRNAs specifically expressed in drought-stressed rice, and 10 lncRNAs specifically expressed in the control group. In addition, after screening four drought-specific genes (Os01g0910800, Os02g0160400, Os05g0586700, and Os07g0188700), we additionally found 16 drought-specific lncRNAs (MSTRG.12273.1, MSTRG.13082.1, MSTRG.19712.1, MSTRG.2089.1, MSTRG.2618.1, MSTRG.27254.1, MSTRG.28695.6, MSTRG.29800.1, MSTRG.30784.1, MSTRG.3087.1, MSTRG.32565.1, MSTRG.35776.1, MSTRG.37520.1, MSTRG.43115.1, MSTRG.43225.1 and MSTRG.7501.1) that had the same target gene Os05g0586700 and were up-regulated. The GO enrichment analysis resulted in Os05g0586700 annotating to the intrinsic components of the membrane (GO:0,031,224) and intracellular membrane-bounded organelle (GO:0,043,231). Therefore, we speculate that Os05g0586700 plays a role in plant membrane repair under drought stress, and the 16 lncRNAs specifically expressed under drought stress may be important regulatory factors of this process.

Active metabolism is the basic response of plants to drought stress [32]. Most of the differentially-expressed lncRNAs identified in our study were also annotated in pathways related to metabolic process. According to our data, Os03g0729000 was significantly up-regulated after drought stress. Furthermore, we inferred by NR annotation that Os03g0729000 was likely to be zinc metalloprotease EGY3, which can promote the stability of chloroplast copper/zinc superoxide dismutase and improve stress resistance when plants are stressed [33]. In addition, we predicted that 28 lncRNAs could target Os03g0729000 (EGY3), and all of them were up-regulated. In response to drought stress, OsCRP1 can generate a large amount of adenosine triphosphate (ATP) in plants by enhancing cyclic electron transport activity, thus improving a plant’s response in drought tolerance [34]. In our study, Os06g0710800 (CRP1) was up-regulated in plants. There were also 34 lncRNAs that predicted Os06g0710800 (CRP1) as targets. Six of these 34 lncRNAs were down-regulated. The specific regulatory mechanism of these six down-regulated lncRNAs need further study. OsbZIP72 can improve drought resistance of rice through ABA signal transduction [35], and overexpression of OsTPKb can alter K^+^ homeostasis in vacuoles and improve drought tolerance in plants [36]. In our study, both Os07g0644100 (bZIP) and Os07g0108800 (TPKb) were up-regulated due to drought stress. We predicted 28 up-regulated lncRNAs targeting Os07g0644100 and 48 up-regulated lncRNAs targeting Os07g0108800. We speculate that there may be a regulatory relationship between them.

miRNA can negatively regulate mRNA through the direct shearing of mRNA and translation inhibition [37]. In our research, a total of 33 drought-related miRNAs were identified. Among them, we found that miR156 was significantly up-regulated in plants under drought stress. Overexpression of miR156 can increase the levels of abscisic acid and antioxidants in plants [38] and affect photosynthesis and photorespiration by silencing SPL13, thereby alleviating the damage caused by drought stress [39]. GO enrichment analysis of its target genes indicated that the terms hormone operation (GO:0,009,914) and transport (GO:0,006,810) were enriched, indicating that our results are consistent with results of previous studies. Overexpression of miR396a has been shown to reduce the water retention capacity and proline content of plants [40]. Our study found that miR396 was significantly down-regulated in Shanlan upland rice under drought stress, which was consistent with previous studies [41]. We analyzed the target genes of miR396 and found that three up-regulated genes, Os01g0133500, Os11g0160100, and Os03g0299800, were enriched to metal ion transmembrane transport activity, transmembrane transport and other pathways. These results suggest that miR396 may play an important role in the ion transport process in plants by regulating Os01g0133500, Os11g0160100 and Os03g0299800. Notably, miR398 was significantly up-regulated in response to drought stress, which is inconsistent with the results of wheat [42], safflower [43] and pea [44]. We speculate that responses to drought may be species-dependent and so they exhibit differences in types and numbers of target genes in miR398 as well as different degrees of gene expression.

Researchers have reported that lncRNA can act as ceRNA to inhibit miRNA function and compete with other targets of miRNA for miRNA [16]. So far, there are no studies on ceRNA in Shanlan upland rice, a typical Hainan upland variety of rice. Based on transcriptome sequencing data, we constructed, for the first time, a ceRNA network for Shanlan upland rice in response to drought stress. In our network, Os02g0662700, Os02g0663100 and Os06g0105350 were annotated as SCL6 by NR annotation. These key genes that respond to drought stimuli together with MSTRG.28732.3 formed a ceRNA network by targeting the same miRNA, miR171. Chlorophyll biosynthesis is inhibited in plants under drought stress [45]. Overexpression of miR171 can increase chlorophyll content in plants [46]. In addition, phytochrome interacting factors PIFs are negative regulators of chlorophyll synthesis, and scarecrow-like protein (SCL) and PIFs control chlorophyll biosynthesis in different but synergistic ways [47]. A large number of studies have shown that miR171 can regulate SCL and play an important role in plant flowering [48], stem branching [49] and callus transformation [50]. Reportedly, miR171 can negatively regulate chlorophyll synthesis by targeting SCL6. In our study, we found that MSTRG.28732.3 may positively regulate SCL6. Therefore, we speculate that SCL6 inhibits chlorophyll biosynthesis in rice and is affected by MSTRG.28732.3. Moreover, miR171 binds to MSTRG.28732.3 to hinder the interaction between MSTRG.28732.3 and SCL6, and miR171 does not directly participate in the synthesis of chlorophyll. We also found that most of the target genes of MSTRG.28732.3 and miR171 annotated to membrane-related entries, such as intracellular membrane-bounded organelle (GO:0,043,231) and intrinsic component of membrane (GO:0,031,224). We speculate that SCL6 is regulated by MSTRG.28732.3 and miR171, playing a role in the formation of chlorophyll membranes, but the specific regulatory mechanism needs further study. Finally, qRT-PCR was used to verify the ceRNA network, and the results were consistent with our predicted regulation mode. The difference Os12g0270300 down-regulated 1.4 times, which was significantly different from the 6.2 times of the sequencing result. We speculated that the difference may be caused by differences between samples.

## Conclusions

We analyzed the expression characteristics of lncRNA in Shanlan upland rice in response to drought stress. Through strand-specific transcriptome sequencing and small RNA sequencing, a series of drought-related lncRNAs, miRNAs, and mRNAs were identified. Through GO enrichment and KEGG pathway analyses of lncRNA target genes, we found that lncRNA played a role in the process of rice resistance to drought stress. Furthermore, MSTRG.28732.3 may act on the common target genes Os02g0662700, Os02g0663100 and Os06g0105350 with miR171, which plays a role in the synthesis of chlorophyll membranes in plants, thereby affecting the ability of plants to resist drought stress. This study provides a theoretical basis for further research on the potential function of lncRNA in plant drought resistance and also provides new gene resources to genetic engineering of crop drought-resistance.

## Methods

### Plant material and drought stress treatment

The Hainan upland variety “Shanchuan 24” was used as the test material. The plant materials were confirmed by a professor Qianhua Yuan (Hainan University, China) and were reserved at the College of Tropical Crops of Hainan University in China. Upland rice seeds of the same size and with their chaff intact were selected and placed in petri dishes. These dishes of seeds were placed in an incubator for 2 days at 35°C to accelerate germination. We filled pots (8.5 cm × 8.5 cm × 9 cm, l × W × H) with clay loam soil to approximately 3/4 of the total volume of the pots. Seedlings that germinated in the petri dishes were selected and planted in soil-filled pots and grown at a room temperature of 25–30°C. The irrigation regime we used maintained a 2-cm layer of water on the surface of the soil in the pots. When the rice grew to the three-leaf and one-heart stage, watering was discontinued to start the drought treatment (D), while the control group (CK) continued to receive watering that maintained the 2-cm layer of water above the soil. Each group had three replicates (CK1, CK2, CK3 and D1, D2, D3). After 15 days of the drought treatment, leaves from each replicate in the control and treatment groups were collected, and 0.5 g of leaf material was taken from each sample and stored at -80°C for RNA extraction, sequencing library construction and further experiments.

### Extraction of total RNA

Total RNA was extracted from plants in the control and drought groups according to the instructions of the RNA Easy Fast Plant Tissue Kit (DP452, Tiangen Company, Beijing). After RNA extraction, quality testing was carried out. First, RNA degradation and contamination were detected on a 1% agarose gel. The purity of RNA was then measured by a nano-photometer (IMPLEN, CA, USA). Finally, RNA concentration and integrity were evaluated using the Qubit2.0 fluorometer (Life Technologies, CA, USA) and Agilent 2100 Bioanalyzer System (Agilent Technologies, CA, USA). After passing the quality detection, RNAs were used to constructe libraries.

### Preparation of strand-specific library and small RNA library

For mRNAs and lncRNAs, a total amount of 1.5 μg RNA per sample were used to construct a strand-specific library. A total of six cDNA libraries (D1, D2, D3 and CK1, CK2, CK3) were constructed from the drought and control groups. First, we used the Ribo-zero™ rRNA Removal Kit (Epicentre Biotechnologies, Madison, WI, USA) to remove rRNA from the total RNA. Then the sequencing library was generated by NEBNextR UltraTM Directional RNA Library Prep Kit for IlluminaR (NEB, USA). The AMPure XP system (Beckman Coulter, Beverly, USA) was used to purify the library fragments. Then 3 μL USER Enzyme™ (NEB, USA) was mixed with size-selected, adaptor-ligated cDNA at 37℃ for 15 min and then PCR was performed with the resulting cDNA. Then, the library quality was evaluated on the Agilent 2100 Bioanalyzer System. The TruSeq PE Cluster Kit V3-cBot-HS (Illumina) was used for clustering from libraries of acceptable quality. Finally, sequencing was performed on the Illumina Novaseq 6000 (Tsingke Biotechnology Co., Ltd, China).

Small RNA libraries were obtained from 3-ug RNA samples, which were constructed by NEBNext® Multiplex Small RNA Library Prep Set for Illumina® (NEB, USA). Library were checked for quality control using the Agilent 2100 Bioanalyzer System, and clustering was conducted. After clustering, sequencing was performed using the Illumina Hiseq 2500 platform (Tsingke Biotechnology Co., Ltd, China).

### Identification of lncRNA and mRNA

Clean reads obtained from the original sequencing data were checked for quality control and compared with the reference genome sequence using HISAT2 [51]. The genome of *Oryza sativa.*IRGSP-1.0 (ensemble, release-42) obtained from an Ensemble database (ftp://ftp.ensemblgenomes.org/pub/release-42/plants/fasta/oryza_sativa/dna/Oryza_sativa.IRGSP-1.0.dna.toplevel.fa.gz) was used as the reference genome. We used StringTie [52] software to assemble the results for comparison and to get mapped reads. The mapped reads were then spliced together and compared with the original genome annotations to look for unannotated transcription regions. Sequences that encoded short peptides with less than 50 amino acid residues or contained only a single exon were filtered out to discover new transcripts and new genes of the species.

LncRNA identification consisted of two parts: basic screening and potential coding-ability screening. The process of basic screening entailed selection of (1) transcripts with class_code of “i”,“x”, “u”, “o”, and “e”; (2) transcripts of ≥ 200-bp length and exon numbers ≥ 2; and (3) transcripts with FPKM ≥ 0.1. We used four different software programs developed to screen for potential coding ability; these were the CPC, CNCI, CPAT, and PFAM protein domain analysis tools [53–56]. In order to visually display results from the four analyses, we constructed a Venn diagram, and the same data identified within the four datasets were used for subsequent lncRNA analysis.

### Identification of miRNA

In order to ensure the accuracy of information analysis, quality control of raw data is required to obtain high-quality sequences (clean reads). To obtain clean reads, we removed unacceptable sequences if a sequence was of low quality, had an unknown base N (N is unrecognizable base) content ≥ 10%, lacked the 3’ linker sequence, or was shorter than 15 nucleotides or longer than 35 nucleotides. Next we filtered out ribosomal RNA (rRNA), transfer RNA (tRNA), small nuclear RNA (snRNA), small nucleolar RNA (snoRNA) and other ncRNA and repetitive sequences to obtain unannotated reads containing miRNA. The Bowtie [57] software was used to align sequences to obtain the location information in the reference genome (*Oryza sativa.*IRGSP-1.0*,* ensemble, release-42), namely mapped reads. Reads from the reference genome were compared with mature miRNA sequences from miRbase (http://www.mirbase.org/), a known miRNA database. Reads whose sequences were identical to those of known miRNAs were considered to be identified as known miRNAs. The miRDeep2 [58] was used to obtain the possible precursor sequence by comparing the location information of reads to the *O. sativa* reference genome. Based on the distribution of reads on the precursor sequence to predict new miRNA.

### Differentially expressed lncRNA, miRNA and mRNA under drought stress

Gene expression is spatio-temporal specific and can be influenced by external stimuli and the internal environment. Genes may express at different levels in response to different conditions (e.g., control vs. treatment, wild-type vs. mutant, different time points, different tissues, etc.). The genes with significantly different expression levels are called differentially-expressed genes. Similarly, transcripts with significantly different expression levels are called differentially-expressed transcripts. In the process of differential-expression gene detection, the screening criteria used to declare differential expression are fold change (FC) and *P*-values. FC is the ratio of expression levels between two samples (groups), and R-packet DESeq is used to calculate FC and *P*-value. The standard values that signify significance are |log2 (FC)|≥ 2 and *P* < 0.05 when screening lncRNA. The screening criteria for differential miRNAs were |log2 (FC)|≥ 1 and *P* < 0.05. For mRNA, the screening criteria were |log2 (FC)|≥ 2 and FDR < 0.05 (we also adjusted mRNA data based on a false discovery rate [FDR]). The FPKM (fragments per kilobase of transcript per million fragments mapped) value was calculated using StringTie [59] to measure lncRNA and mRNA expression levels. The expression levels of miRNA in each sample were obtained, and the TPM (transcripts per kilobase of exon model per million mapped reads) algorithm was used to normalize the expression levels [60]. Then hierarchical cluster analysis was performed for differentially-expressed lncRNAs, miRNAs and mRNAs.

### Target gene prediction and functional annotation

LncRNA cis-target genes are mainly predicted based on the positional relationship between lncRNA and target genes. We found adjacent genes within 100 kb upstream and downstream of lncRNA as cis-target genes of lncRNA by Perl script [61]. Trans-target genes interact with lncRNA by base complementary pairing, and lncTar, a target gene prediction tool, was used to predict them [62]. The TargetFinder software was used to predict differentially-expressed miRNA target genes.

For the functional annotation of differential genes, we used the R package topGO [63] to perform GO analysis of the differentially-expressed genes (http://www.geneontology.org/). BLASTX was used to search the NR database (ftp://ftp.ncbi.nih.gov/blast/db/) for association information about genes. We used the KOBAS [64] software to detect the degree of statistical enrichment of differentially-expressed genes in the KEGG pathway.

### ceRNA network construction and analysis

First, target genes of miRNA, mRNA and lncRNA were predicted by TargetFinder, namely, the relationship pairs of miRNA-mRNA and miRNA-lncRNA target genes. Then, ceRNAs were screened by the ceRNA-score principle [65, 66]. The standard were that lncRNAs and mRNA with the same numbers of miRNAs greater than three, *p* < 0.05 and FDR value < 0.1. FDR was obtained by correcting *p*-values using the p.adjust function in R. The lncRNAs, miRNAs and mRNA in the relationship pairs were compared and screened with the differentially-expressed lncRNAs, miRNAs and mRNA in rice under drought stress. Then, all the differentially-expressed lncRNA-miRNAs, miRNA-mRNA and miRNA-mRNA relationship pairs were obtained. Finally, the relationship pairs with negative correlation between miRNAs and targets of miRNAs and positive correlation between miRNAs targets were selected, and these relationship pairs were combined to obtain ceRNA network. The data were used to visualize using the Cytoscape3.8.2 software.

### The correlation between miRNA and ceRNA expression under drought stress was verified by qRT-PCR

Real-time quantitative polymerase chain reaction (qRT-PCR) was used to analyze the relative expression levels of lncRNA, miRNA and mRNA in Shanlan upland rice subjected to drought stress for 0 and 15 days. Plant total RNA was extracted using the RNA Easy Fast Plant Tissue Kit (DP452) from Tiangen Biotech Co., Ltd. (Beijing, China). The SnapGene (v3.2.1) software was used to design lncRNA and mRNA primers. MiRNA reverse transcription and qPCR primers were designed using miRNA Design (www.vazyme.com) launched by Vazyme Biotech Co., Ltd. (Nanjing, China). Quantitative analysis was performed using an ABI Quant Studio 6 Fluorescence Quantitative PCR System (Applied Biosystems by Life Technologies, United States). Relative RNA expression levels were calculated using the 2^−△△CT^ method [67]. T-test was used to check the significance of samples between different treatments by Excel with *P* < 0.05 considered to be significant difference and *P* < 0.01 considered to be extremely significant difference [68]. Three technical replicates were set up for each group of data, and the standard deviations were calculated. β-actin was used as the internal reference gene of lncRNA and mRNA, and U6 was the internal reference gene of miRNA. All primer sequences are listed in supplemental Table S00^3^.

## Supplementary Information

**Additional file 1: Table S001.** lncRNA specifically expressed in drought.

**Additional file 2: Table S002.** lncRNA specifically expressed in CK.

**Additional file 3: Table S003.** The primers used for qRT-PCR.

**Additional file 4: Table S004.** List of differentially expressed lncRNAs from the two treatment groups.

**Additional file 5: Table S005.** List of differentially expressed miRNAs from the two treatment groups.

**Additional file 6: Table S006.** Function annotion of lncRNAs cis-target gene.

**Additional file 7: Table S007.** Function annotion of lncRNAs trans-target gene.

**Additional file 8: Table S008.** Function annotion of miRNAs target gene.

**Additional file 9: Table S009.** Function annotion of mRNAs.

**Additional file 10: Table S010.** The relationship of miRNA with lncRNA and mRNA.

**Additional file 11: Table S011.** The relationship of lncRNA with Mrna.

**Additional file 12: Table S012.** Accession number of SRA.

## Data Availability

All data analyzed during this study are provided in this published article and additional files. The sequence data of this study have been deposited into SRA database (https://www.ncbi.nlm.nih.gov/sra), accession number in Supplemental table S0^12^.

## Abbreviations

lncRNA: Long non-coding RNA
miRNA: MicroRNA
ceRNA: Competing endogenous RNA
DRIR: Drought-induced RNA
ABA: Abscisic Acid
lincRNA: Long intergenic lncRNA
MRE: MicroRNA response element
IAA: Indoleacetic acid
circRNA: Circular RNA
GO: Gene Ontology
KEGG: Kyoto Encyclopedia of Genes and Genome
SCL: Scarecrow-like protein
SCL6: Scarecrow-like protein 6
NR: NCBI non-redundant protein sequences
siRNA: Short interfering RNA
piRNA: PIWI-interacting RNA
D: Drought
CK: Contrast
FPKM: Fragments Per Kilobase of transcript per Million fragments mapped
FC: Fold Change
rRNA: Ribosomal RNA
snoRNA: Small nucleolar RNA
snRNA: Small nuclear RNA
ncRNA: Non-coding RNA
tRNA: Transport RNA
ROS: Reactive oxygen species
FDR: False Discovery Rate
